# Evaluating non-responders of a survey in the Swedish fracture register: no indication of different functional result

**DOI:** 10.1186/s12891-017-1634-x

**Published:** 2017-06-28

**Authors:** Hans Juto, Mattis Gärtner Nilsson, Michael Möller, David Wennergren, Per Morberg

**Affiliations:** 1 Department of Surgical and Perioperative Science (Orthopedics), Sunderby Research Unit, Umeå University, Umeå, Sweden; 2 Department of Orthopedics and Trauma, Sahlgrenska University Hospital, Gothenburg/Mölndal, Sweden

**Keywords:** Swedish Fracture Register, Non-responders, PROMs, Survey, SMFA

## Abstract

**Background:**

The Swedish Fracture Register (SFR) currently contains information on more than 190,000 fractures. Patient Reported Outcome Measures (PROMs) are used for monitoring functional results after fracture treatment. One weakness, as in many surveys, is a low response rate. The aim of the current study was to examine if non-responders of a survey in the SFR differ in PROMs scores, how age and gender influence the response rate and reasons for not responding.

**Methods:**

Patients with fractures of radius, ulna or humerus between June and August 2013 and registered in the SFR were included in the study. The non-responders to both the pre-injury and the 1-year survey were contacted by phone and reminded to reply. A comparison of the results of both EQ-5D and Short Musculoskeletal Function Assessment (SMFA) could be made between the responders after a phone reminder and the initial responders. The response rate for the register as a whole was extracted in order to identify how age and gender affect the response rate.

**Results:**

Three hundred seventeen of the patients included in the study responded initially. After phone reminder another 94 patients answered the pre-injury survey. Two hundred sixty eight responded initially to the 1-year follow-up survey and 42 after phone reminder. No significant difference was identified in the score of the pre-injury survey between initial responders and responders after phone reminder neither in the EQ-5D nor in the Short Musculoskeletal Function Assessment (SMFA). Regarding the 1-year survey, responders after a phone reminder reported a significantly better outcome in crude data of SMFA score. This difference disappeared after controlling for confounding factors through case control matching. The highest response rate to PROMs in the SFR was among females in the age range 60–69 years.

**Conclusion:**

This study indicates that both in the preinjury survey as well as in the 1-year survey the non-responders in the SFR report similar function compared to the initial responders. Age and gender of patients affect the response rate of the survey which needs to be taken into consideration in analysis of data from the SFR.

## Background

The Swedish Fracture Register (SFR) is a non-mandatory register where information about patients, the type of trauma, fracture classification according to AO/OTA, treatments and patient reported outcomes are recorded [1]. Only injuries of patients with a Swedish personal identity number are registered. About 60% of Swedish hospitals treating fractures participate in the register. More than 190,000 fractures have been recorded and the number increases by approximately 4000–5000 per month. The five most frequent fractures in the SFR are distal radius fractures, trochanteric and cervical femoral fractures, proximal humeral fractures and ankle fractures. Studies on the validity of the classification of fractures in the SFR have been conducted [2, 3].

The SFR uses the Short Musculoskeletal Function Assessment (SMFA) and the EQ-5D as Patient Reported Outcome Measures (PROMs). Both SMFA and EQ-5D are well validated tools for assessment of function. The SMFA consists of 46 questions; 12 of the questions concerning how much the patient was bothered by different problems and 34 questions concerning the function of the patient. The results are summarised in a bother index and a dysfunction index. The dysfunction index can further be divided into four different categories: daily activities, function of the arm and hand, emotional status and mobility. Each category’s score is transformed into one of the ranges from 0 to 100, with a low score representing a higher function [4]. Ponzer et al. translated and adapted the American version of the SMFA for Swedish use in 2003. They conclude that the SMFA is easy to use, has good reliability and is sensitive to changes in function [5].

The SFR send two surveys to patients after suffering from a fracture. One immediately after the injury, where the patients are asked to evaluate their function before the trauma (pre-injury survey). A second survey is sent, one year after injury, to those who answered the pre-injury survey. A reminder is routinely sent to those who did not reply to the first mailed survey.

The SFR gives unique possibilities in research on fracture treatment when combining large amount of data even on less common fracture subgroups with patient reported outcome. Low response rate can however be a problem with patient involvement in medical registries. A systematic review of 219 studies report an average response rate of approximately 60% among mail surveys published in medical journals [6]. Surveys after elective orthopedic surgery, on the other hand, appear to have a somewhat higher response rate [7–11]. Factors influencing the response rate have been well studied. Short questionnaires, monetary incentives, clinical follow-ups and interesting content in questionnaires increase the response rate [12].

Loss to follow-up and low response rate may introduce bias to a survey and affect the validity of a study [13–16]. This problem has been addressed in several earlier studies and non-responders or patients who are lost to follow-up may report poorer functional results than initial responders. Two different studies on follow-up after primary total knee arthroplasties showed that non-responders to posted mail questionnaires had a significantly worse function compared to the initial responders [10, 11]. Similar results have been shown on loss to follow-up after both hip arthroplasty and rotator cuff tears [17, 18]. No difference in outcome between initial responders and non-responders were on the other hand shown in studies on shoulder arthroplasty and degenerative lumbar spine surgery [8, 9].

The aim of this study was to examine if non-responders of a survey in the SFR differ in PROMs score in both day 0 and 1-year survey from patients who initially responded. We also examined whether age and gender influenced the response rate, and the reasons for not responding to the survey.

## Methods

The study included all patients who were injured between June to August 2013 with fractures of the radius, ulna and humerus (ICD-10 codes: S42.20–42.99 and S52.00–52.99), treated at the Department of Orthopedics and Trauma at Sahlgrenska University Hospital in Gothenburg or Sunderby Hospital in Luleå, Sweden, and registered in the SFR. There were no specific exclusion criteria.

At first a follow-up of non-responders to the pre-injury survey was conducted. Patients from which the SFR had not received a completed survey after a routine reminder were identified. Three attempts were made to reach them by phone. The initial non-responders who were reached by phone were interviewed and asked how satisfied they were with the received healthcare, why they had not answered the initial survey and if they could answer it if a new one was sent. A new survey was only sent to those who were reached by phone and accepted to participate. Responding after phone reminder was defined as when a survey arrived from a patient after a phone interview had been conducted. Patients still not responding after interview or attempt of phone reminding was defined as eventual non-responders.

The 1-year follow-up survey was mailed from the SFR to patients who had answered the day 0 survey according to ordinary routine. This included both the initial responders and those responding after phone contact from the pre-injury survey. As for the day 0 survey a postal reminder was routinely sent to those who did not answer the initial mailed survey. The method for the 1-year follow-up was the same as the day 0 survey. This enabled a comparison of the PROMs scores between the initial responders and those responding after phone contact from both the survey sent immediately after the fracture and the survey sent one year after the fracture.

For information about the response rate in general, all recorded surveys and recorded fractures from July 2013 to June 2014 were extracted from the SFR in aggregated form and compared according to gender and age. Age was divided into groups with 10 years age span (20–29 etc.) as well as below 20 and above 90.

As outcome for the study we used the EQ-5D and all parts of the SMFA. We also compared the satisfaction with received care from responders after the phone reminder to non-responders after the phone reminder. Also as outcome we asked the interviewees to grade their received healthcare on a scale from one to ten, where 10 represented “very pleased”. The power calculation was made on dysfunction index of the SMFA. It estimated that for the detection of a difference of 5 point and with a power of 0.8, 85 and 255 surveys respectively in the different groups were needed.

Because of the skewed distribution of results in both the EQ-5D and the SMFA we used logistic regression to consider other affecting variables. We defined a low functional outcome as a higher score than median or mean value of dysfunction index as well as arm/hand function sub-index of the SMFA. We then dichotomized the score and examined the odds ratio for a low functional result.

The explaining variables used in the logistic regression model were: survey before and after phone reminder, age, gender, case mix, open fracture, earlier and later fracture registered in the SFR (within one year before and after) and day 0 SMFA dysfunction index (only used on 1-year PROM). The fractures were divided into six groups where each included the most common ICD-10 diagnosis, multiple fractures and in the last group the least common ICD-10 diagnosis together. The proportion of fractures in the six groups was stated as case mix. Multiple fractures was defined as more than one ICD-10 diagnosis of the same injury. The non-significant variables in the logistic regression model was excluded one by one and only the significant ones are reported.

Case control matching were also used to handle confounding factors. The significant explaining factors from the logistic regression model were used for the matching. In the results of the day 0 survey age, earlier registered fracture and case mix were taken into account when matching. In the 1-year survey age, case mix and the SMFA dysfunction index from day 0 was considered. From the day 0 survey 89 matched pairs could be created and from the 1-year survey, 36 matched pairs.

The non-parametric Mann-Whitney U test was used in the comparison of scores between the two groups and the Pearson Chi-squared test was used for analysis of distribution of proportions. For the analysis on the proportion of females in different groups linear-by-linear association was used because of the falling tendency. The received care data and age distribution was less skewed and therefore the Student’s t-test was regarded robust enough to be used. One-way ANOVA was used for comparison of numerical data between more than two groups. Missing data was not replaced in the analysis. Statistical significance was set at *p* ≤ 0.05. Statistical analysis as well as case control matching was performed by using IBM SPSS version 23.

## Results

### Demography

Six hundred thirteen consecutive patients were included in the study. The mean age of all the patients in the study was 60 years and 402 (66%) of the patients were female. Twelve different fractures according to ICD 10-diagnosis and also cases of multiple fractures of the same injury were included. The most common fractures were distal radius (48%), proximal humerus (20%), proximal radius (8.8%), multiple fractures (6.9%), and diaphyseal humerus fracture (3.9%). The group of the least common fractures constituted 13%. 14 (2.3%) of the patients had an open fracture, 17 (2.8%) had a fracture registered in the SFR the year before this injury and 32 (5.2%) had another fracture within one year after.

The mean age was 62 years in the initial responders groups of both the day 0 and the 1-year survey. This mean age was slightly higher compared to the mean age in responders after phone reminder and the eventual non-responders but the age difference was only statistically significant for the day 0 group. There were also significantly more females among the initial responders than in the other groups in the 1-year follow-up. No significant difference could be detected in the case mix between the groups. The number of open fractures was too small to be statistically analysed. After case control matching the difference in age disappeared as well as the difference in case-mix between the initial responders and the responders after phone reminder (Table 1).

**Table 1:** Demography of the included patients and fractures in both the day 0 (pre-injury) and the 1-year follow-up before and after case control matching. One-way Anova was used for statistical analysis of mean age and Pearson’s Chi-square test for distribution

		Initial responders	Responders after phone reminder	Eventual non-responders	*p*-value
Demography crude data					
Day 0 survey	Number	317	94	202	
	Mean age (sd)	62.2 (19.0)	56.4 (20.3)	58.5 (23.5)	0.026
	Female	214 (68%)	62 (66%)	126 (62%)	0.235 (a)
	Fracture before (b)	8 (2.5%)	3 (3.2%)	6 (3.0%)	0.922
	Case mix (c)	48/19/7/6/4/16	52/16/13/4/4/11	44/22/10/9/4/11	0.330
1-Year survey	Number	268	42	86	
	Mean age (sd)	61.6 (18.0)	58.8 (19.5)	56.0 (22.4)	0.056
	Female	189 (71%)	27 (64%)	51 (59%)	0.048 (a)
	Open fracture	8 (3.0%)	0 (0.0%)	0 (0.0%)	- (d)
	Fracture after (e)	15 (5.6%)	0 (0.0%)	1 (1.2%)	0.071
	Case mix (c)	47/19/9/6/4/16	57/17/10/7/2/7	56/12/8/5/5/15	0.785
		Initial responders	Responders after phone reminder	*p*-value	
Demography data after case control matching					
Day 0 survey	Number	89	89		
	Mean age (sd)	57.1 (20.0)	56.7 (19.7)	0.889	
	Female	48 (54%)	59 (66%)	0.242	
	Fracture before (b)	0 (0.0%)	(0.0%)	- (d)	
	Case mix (c)	55/15/12/3/2/11	55/15/12/3/2/11	1.000	
1-Year survey	Number	36	36		
	Mean age (sd)	56.6 (19.7)	56.8 (19.8)	0.957	
	Female	23 (64%)	27 (64%)	0.971	
	Open fracture	2 (5.6%)	0 (0.0%)	- (d)	
	Fracture after (e)	8 (22.2%)	0 (0.0%)	0.001	
	Case mix (c)	64/19/6/3/3/2	64/19/6/3/3/2	1.000	

^a^Linear-by-linear association used because of the falling tendency

^b^Cases with another fracture registered in the SFR within one year before the fracture

^c^Distribution in percentage of the most frequent diagnosis (ICD-10) in the study (S52.5/S42.2/S52.1/multiple fracture/S42.3/every other)

^d^Statistical analysis cannot be done due to small number

^e^Cases with another fracture registered in the SFR within one year after the fracture

### Day 0 survey

**Fig. 1:**
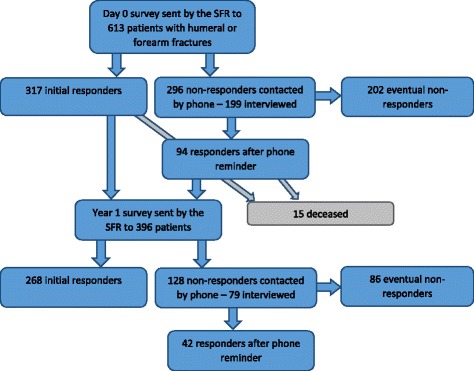
Flow chart of responders and non-responders to the day 0 (pre-injury) and 1-year surveys included in the study

In the pre-injury (day 0) survey, 317 patients initially responded to the survey sent by the SFR. An attempt was made to contact the remaining 296 patients by phone. In 35 cases a valid phone number could not be obtained. Forty eight did not answer to any of the attempts and 14 could not be interviewed for other reasons (illness, living in a nursing home etc.). One hundred ninety nine of the initial non-responders were reached and asked to participate and 94 patients responded to a new survey after the phone contact. Two hundred two patients never responded to the survey (Fig. 1). Responders after phone reminder reported a mean value of 0.845 in the EQ-5D compared with 0.829 in the initial responders group. In the SMFA dysfunction and bother index a mean value of 13.1 and 12.6 respectively were reported by the responders after phone reminder compared to 12.8 and 11.5 for the initial responders. None of the results from the PROMs were significantly different. After compensation for age, earlier fracture and ICD-10 diagnosis with case control matching there was still no significant difference between PROMs scores of initial responders and responders after reminder. (Table 2). When using logistic regression is age the only factor affecting all tested scores. There are however some single cases with significant factors affecting the result. Among others, on median of dysfunction index, when responding after reminder is also a significant factor (Table 4).

**Table 2:** PROMs result of day 0 (pre-injury) survey when comparing the initial responders and the responders after phone reminder both before and after case control matching. Mann-Whitney U-test was used for statistical analysis of score value

		EQ-5D	SMFA – Dysfunction index					SMFA – Bother index
				Daily Activity	Emotional	Arm/Hand Function	Mobility	
Day 0 Survey crude data								
Initial responders	mean	0.829	12.8	14.2	19.5	7.5	11.3	11.5
	median	1.0	4.0	0.0	14.3	0.0	2.8	4.2
Responders after phone reminder	mean	0.845	13.1	14.3	20.1	6.6	12.2	12.6
	median	1.0	6.6	2.5	14.3	0.0	2.8	4.2
	*p*-value	0.846	0.477	0.675	0.569	0.963	0.446	0.580
Day 0 Survey after Case Control Matching								
Initial responders	mean	0.851	11.8	12.1	20.6	5.8	10.3	12.6
	median	1.0	3.7	0.0	14.3	0.0	2.8	6.3
Responders afterphone reminder	mean	0.842	13.2	14.4	20.3	6.8	12.5	16.7
	median	1.0	6.6	2.5	16.7	0.0	2.8	12.5
	*p*-value	0.357	0.544	0.374	0.960	0.415	0.405	0.472

### One year survey

In the 1-year survey, 268 patients responded initially to the survey sent by the SFR. Fifteen of the patients who answered the day 0 survey had died at the time of the 1-year follow-up. An attempt was made to contact the remaining 128 patients by phone and 79 could be reached and interviewed. In nine cases a valid phone number could not be obtained. Twenty seven did not answer at any of the attempts and 13 could not be reached because of other reasons. Forty two of the 79 patients answered the questionnaire after phone contact whereas 86 still were non-responders for the 1-year survey (Fig. 1). In the crude PROMs score of 1-year survey, the EQ-5D demonstrated no significant difference between initial responders and responders after reminder. In the dysfunction index of the SMFA the responders after phone reminder reported a mean score of 10.2 and the initial responders 15.6. This was a significantly better functional result of the responders after reminder. Significant differences were observed in daily activity, emotional and arm/hand function but not in mobility. The significant difference disappeared after case control matching (Table 3). In the logistic regression model, age, preinjury dysfunction index score and case mix, show significant effect on the result (Table 4).

**Table 3:** PROMs result of 1-year survey when comparing initial responders and responders after phone reminder both before and after case control matching. Mann-Whitney U-test was used for statistical analysis of score value

		EQ-5D	SMFA – Dysfunction index					SMFA – Bother index
				Daily Activity	Emotional	Arm/Hand Function	Mobility	
One year Survey crude data								
Initial responders	mean	0.786	15.6	16.8	23.5	10.5	12.9	14.9
	median	0.796	10.3	7.5	21.4	3.1	2.8	8.3
Responders after phone reminder	mean	0.795	10.2	12.5	17.9	5.7	7.4	12.2
	median	0.796	2.9	0.0	14.3	0.0	0.0	4.2
*p*-value		0.388	0.020	0.018	0.050	0.027	0.080	0.067
One year Survey after Case Control Matching								
Initial responders	mean	0.849	9.4	8.5	19.2	5.4	7.2	8.8
	median	0.796	6.3	2.5	14.3	3.1	0.0	6.3
Responders after phone reminder	mean	0.834	8.3	8.1	16.8	4.7	5.1	9.4
	median	0.805	2.9	0.0	8.9	0.0	0.0	0.0
*p*-value		0.566	0.094	0.213	0.258	0.060	0.285	0.154

**Table 4:** Statistical significant variables affecting the risk of low functional result at pre-injury and 1-year PROMs. Logistic regression was used with mean as well as median value on SFMA dysfunction index and arm/hand sub index as cut off on dichotomized data

*Preinjury Survey*	Variable	Odds Ratio	95% CI
*Arm/hand mean*	Age	1.06^a^	1.04–1.09
	ICD-10 (S42.3)	5.25^b^	1.39–19.8
*Arm/hand median*	Age	1.08^a^	1.06–1.10
*Dysfunction mean*	Age	1.05^a^	1.03–1.07
	Earlier fracture	5.22	1.09–25.1
*Dysfunction median*	Age	1.05^b^	1.04–1.06
	Responding after phone reminder	1.87	1.10–3.18
*One year Survey*	Variable	Odds Ratio	95% CI
*Arm/hand mean*	Age	1.03^a^	1.003–1.05
	Preinjury SMFA dysfunction	1.08^a^	1.06–1.11
	ICD-10 (multipel)	3.51b	1.03–12.0
*Arm/hand median*	Age	1.03^a^	1.01–1.05
	Preinjury SMFA dysfunction	1.09^a^	1.06–1.12
	ICD-10 (S42.3)	28.0b	2.63–298
*Dysfunction mean*	Age	1.03^a^	1.01–1.05
	Preinjury SMFA dysfunction	1.12^a^	1.08–1.15
	ICD-10 (S42.3)	25.1^b^	3.33–189
	ICD-10 (every other)	2.58^b^	1.05–6.37
*Dysfunction median*	Age	1.02^a^	1.001–1.03
	Preinjury SMFA dysfunction	1.09^a^	1.06–1.12
	ICD-10 (S52.1)	4.51^b^	1.23–16.5
	ICD-10 (S42.3)	16.5^b^	1.69–161

^a^For every year/point higher age/score

^b^S52.5 used as a reference

### Phone interviews

The mean scores of satisfaction with received care in patients contacted for phone interview varied between 6.4 and 8.4 (range 1–10) in responders vs non-responders in the day 0 and the 1-year follow-up. No significant differences were observed between the groups (Table 5). On the question, why the patient had not initially answered the survey the most common stated reasons were “had not received the survey” (32%), “lack of time” (23%) while “dissatisfaction with received care” or “language problem” were stated by 1% respectively (Table 6).

**Table 5:** Satisfaction with the received care in patients contacted for phone interview on a scale from 1 to 10 with 10 as very pleased. Student’s t-test was used

	Pre-injury Survey		1 Year Survey	
	Number	Mean value	Number	Mean value
Responder after phone reminder	94	7.3	42	8.4
Interviewed eventual non-responders	103	6.4	35	7.4
*p*-value		0.062		0.096

**Table 6:** Stated reason for not initially answering the survey when reached by phone in both follow-up on the pre-injury and the 1-year survey

Number of interviews	Not received the survey	Have already answered the survey	Lack of time	Not interested	Dissatisfaction received care	Problem language	Illness
278	32%	22%	23%	11%	1%	1%	9%

### Overall response rate

**Fig. 2:**
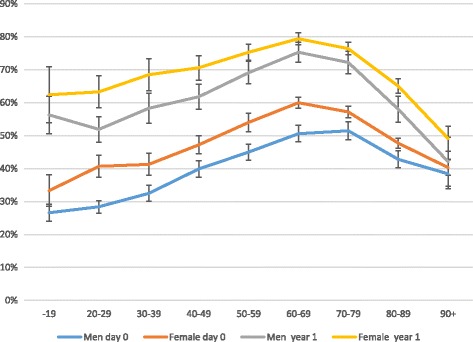
Response rate of all registered patients and injuries in the SFR from July 2013 to June 2014 for both day 0 and 1-year survey according to age and sex with 95% Confidence Intervals

The overall response rate for the 30,275 patients injured between 1 July 2013 and 31 June 2014 and registered in the SFR was 46% for the initial preinjury (day 0) survey. Of those who answered the preinjury survey, 68% also answered the 1-year follow-up survey. Men had a lower response rate with 39% at day 0 survey and 63% at 1-year follow-up, compared to 51% and 71% for women respectively. The highest response rates were found in patients between 60 and 69 years of age in all categories except for men at day 0 survey. The lowest response rates were found among the youngest as well as among the oldest patients. The response rate decreases especially among the oldest in the 1-year follow-up survey (Fig. 2).

## Discussion

When comparing crude PROMs scores of initial responders and responders after phone reminder, no differences were demonstrated for the results of the pre-injury surveys, neither for the EQ-5D nor the SMFA. In contrast, the 1-year follow-up showed indication of better functional result in the phone reminder group. The differences were significant for the dysfunction index as well as for the daily activity, the emotional and the arm/hand function sub-indices. However this difference is probably due to the difference in constitution between the groups. In the logistic regression model, age, preinjury SFMA dysfunction index and case mix, affect the result of the 1-year SMFA score but not if a survey was sent initially or after reminder. When controlling for them in a case control matching the difference in crude SMFA score disappears.

Even though crude PROMs scores at day 0 survey do not differ between initial responders and responders after reminder, this could be false and there could be a real difference hidden behind the age difference. After controlling for confounding factors with case control matching there is still no significant difference. When using logistic regression, in one case, responding after reminder can be seen as an independent factor.

When we interpret the overall results, we do not see any obvious difference between PROMs results of initial responders and non-responders, neither in the day 0 nor the 1-year survey. Former similar studies on follow-up after elective orthopedic surgery have in some studies shown no difference between responders and non-responders and in some a worse functional result for non-responders. [8–11, 17, 18]. Our result of no difference in patient reported outcome is in concordance with those of Polk et al. and Solberg et al. [8, 9].

A weakness in the study to consider is that only one third of the initial non-responders eventually sent in their surveys after phone contact. This could still lead to a selection bias where the eventual non-responders are patients with factors which link functional results to non-responding. For example dissatisfaction with received care in patients with bad results. Another potential bias with the study is that the intervention, the phone reminder, itself effects the result. Interview bias is hard to avoid but we tried to minimise the intervention by keeping the phone call short, neutral and not offering any consideration to convince the initial non-responder to respond. Another issue is that there is the quite low power after case control matching leading to the possibility of a type 2 error. Nevertheless indicate this study that there are no difference in functional results between initial responders and non-responders to a survey in the SFR.

We furthermore demonstrate that the response rate depended on the patient’s age and gender. The highest response rate was found among women between 60 and 69 years of age. A small difference in mean age between responders and non-responder but no significant difference between genders were shown in a study on the Swedish Hip Arthroplasty Register [7]. Solberg et al. as well as Polk et al. found that the response rate increased with age [8, 9]. We agree with this, but we also observed that the response rate tends to decrease when the patient is over the age of 80. In this age group of very elderly, many have co-morbidities that are likely to influence the ability to participate in the survey. These patients are probably not included in the other studies as comorbidities in combination with high age make them less likely candidates for elective surgery. We also believe it must be taken into consideration when analysing PROMs data that the response rate is considerably lower for younger patients with usually higher functional demands.

Murray et al. conclude that vigorous attempts must be made to minimise loss to follow-up and to reduce the risk of bias [18]. In a registry this is difficult because of the large number of patients included and the costs. Administration of PROMs is the largest costs linked to the SFR. For patients registered in the SFR one postal reminder is sent whereas reminders of any kind will probably increase the reply rate, but the costs cannot be justified.

Response rates to surveys, in for example an arthroplasty register are usually considerably higher than in the SFR. One of the reasons for this may be that arthroplasty patients are usually relatively healthy and they are in the age groups most likely to answer a survey. In the SFR, it is the participating orthopedic departments who administer the surveys and there are obvious differences between different departments in response rates. Over time, as the departments improve their routines, the response rate will most likely rise. However, a fracture register will probably never achieve the same response rate even under optimal conditions, as surveys after elective surgery.

In the phone interviews, two of the most common stated reasons for not initially answering the survey were “I did not receive the survey” or “I have already sent back the survey”. Even if this, in some cases is correct, we do not believe the extent of the problem is with the postal service, but see this as an evasion. Dissatisfaction with received care was expected to be a frequent reason for not responding as earlier described but neither the questionnaire nor the grading indicated it [11, 18]. We interpret the main reason for not participating among younger patients is a lack of priority, and among the very elderly, illnesses.

## Conclusion

This study indicates that both in the preinjury survey as well as in the 1-year survey the non-responders in the SFR report similar function compared to the initial responders. Age and gender of patients affects the response rate of the survey which needs to be taken into consideration in analysis of data from the SFR.

## Abbreviations

AO/OTA: Arbeitsgemeinschaft fur osteosynthesefragen (Association for the Study of Internal Fixation)/ (American) orthopedic trauma association
EQ-5D: European quality of Life-5 dimensions
ICD-10: International statistical classification of diseases and related health problems *(10th edition*)
PROM: Patient reported outcome measures
SFR: Swedish Fracture Register
SMFA: Short musculoskeletal functional assessment
